# Variation of Critical Water Pressure for Hydraulic Fracturing in Cement Mortar under Sulfate Attack

**DOI:** 10.3390/ma15041595

**Published:** 2022-02-21

**Authors:** Detan Liu, Hongwei Zhang, Zhenzhong Shen, Liqun Xu, Qiong Wu, Yidong Ge

**Affiliations:** 1 College of Water Conservancy and Hydropower Engineering, Hohai University, Nanjing 210098, China; liudetan1993@163.com (D.L.); zhzhshen@hhu.edu.cn (Z.S.); 2 Datang Hydropower Science & Technology Research Institute Co., Ltd., Nanning 530007, China; 3 Shandong Survey and Design Institute of Water Conservancy Co., Ltd., Jinan 250013, China; wqdeyouxiang2022@163.com; 4 Huaian Water Conservancy Survey and Design Research Institute Co., Ltd., Huaian 223010, China; gyd18762039859@163.com

**Keywords:** cement mortar, critical water pressure, hydraulic fracturing, sulfate attack, evolution model

## Abstract

Hydraulic fracturing may be induced easily in a cement-based structure in a sulfate-rich environment, which threatens engineering safety. In order to investigate the evolution of critical water pressure, a series of hydraulic fracturing tests and splitting tensile strength tests on the cement mortar under different sulfate-exposure periods are performed. The critical water pressure of the cement mortar under sulfate attack experiences an initial increase stage and a subsequent decrease stage. A stress intensity factor is modified by two proposed damage variables which are crack length and fracture stress. Then, the relationship between the critical water pressure and the tensile strength is established. Moreover, an evolution model of the critical water pressure is proposed, which reveals that the matrix tensile strength and porosity of cement mortar strongly affect the critical water pressure evolution. Additionally, an empirical formula is suggested to describe the critical water pressure evolution of the cement mortar under sulfate attack, and its validity is verified by experimental results.

## 1. Introduction

Cement mortar material has been applied widely in hydraulic engineering (dams, offshore platforms, and hydraulic tunnels) due to its low price, high strength and good durability. However, as a building material, the cement mortar is easy to produce cracks due to the influence of the designed mix proportion, temperature and construction technology factors. When hydraulic structures unfortunately suffer a high water pressure, water inflow into the crack faces can further promotes the existing crack to develop a major crack, which weakens the bearing capacity of hydraulic structures [1]. Hence, it becomes important to understand the phenomenon of hydraulic fracture that happens in cracks in cement mortar materials for the safety evaluation of hydraulic structures. For this purpose, considerable research on hydraulic fracturing of the cement mortar has been carried out. For instance, Bruwiler and Saouma [2] employed wedge splitting specimens to investigate the distribution of water pressure within a concrete crack. Their tests indicated that the water front along the propagating crack lags behind the extension of the crack tip. Using this concrete specimen, Slowik and Saouma [3] found that the cracking opening rate has a major impact on the water pressure distribution in the concrete crack under dynamic loading. Furthermore, Chen et al. [4] determined a water pressure distribution model and failure patterns in a concrete crack via an improved hydraulic device and wedge-splitting tests. And the prediction model of critical water pressure and the hydraulic fracturing mechanism of concrete specimen were also discussed by utilizing the cylinder specimen with an embedded crack [5] and the central-precrack cube specimen [6]. For the numerical simulation of hydraulic fractures in concrete structures, several studies have been reported widely [1,7]. However, none of the above research considered the influence of chemical erosion on the hydraulic fracturing of cementitious materials.

The long-serving environment of hydraulic structures is very complex, it often has chloride ions, sulfate ions, and sodium ions, hydrogen ions and so on. Sulfate ion as a common corrosive ion, which can result in undermining the structural integrity and shortening service life of hydraulic structures [8,9,10]. When the sulfate ions penetrate into cement mortar and react with the hydration products to produce expansive products. Under the action of this expansive products, the micro-cracks will be occurred. The development of micro-cracks will accelerate the sulfate ions to penetrate into the cement mortar materials, resulting in the deterioration of the hydraulic structures [11]. Thus, it is necessary to study the behavior of cement mortar material under sulfate attack, for minimizing the degradation effect of sulfate attack. Recently, several researchers have studied the durability of cement mortar materials under sulfate attack. For instance, Yu et al. [12] revealed the evolution of the compressive strength, elastic modulus and water permeability coefficient of cement mortar subjected to sulfate attack via a series of tests. Yao et al. [13] performed the three-point bending tests to investigate the variation law of fracture toughness of cement mortar due to sulfate attack, and a chemo-mechanical evolution model was established to describe the evolutionary behavior of fracture toughness of cement mortar caused by sulfate attack. Ouyang et al. [14] studied the evolution of surface hardness of concrete under sulfate attack and found that the water-to-cement ration and the concentration of sulfate solution significantly affect the attenuation tendency of the surface hardness. Zhang et al. [15] showed that, under direct shearing, the failure criterion of concrete materials is obviously affected by external sulfate attack. Chen et al. [16] pointed out that the average size of microvoids in cement mortar materials controls the damage evolution of specimens. With the application of scanning electron microscopy and X-ray diffraction in microstructural analysis, the influence of different sulfate solution on the damage degradation on concrete was investigated [17,18], and the evolution of microstructure in cement mortar caused by the nucleation and growth of gypsum and ettringite, was also investigated to understand its degradation mechanism [12,14,19]. Furthermore, the sulfate resistance of cement mortar has been studied using fly ash, silica fume, blast furnace slag, rice husk ash, and other new components [20,21,22]. In theoretical studies, the diffusion model of sulfate ions [11], the chemo-mechanical damage constitutive model [23], Wu’s plastic-damage model [24] and the transport-chemical-mechanical model [25] were established. Although some research regarding the performance change of cement mortar due to sulfate attack has been carried out, the damage evolution of the hydraulic fracturing performance of cement mortar subjected to sulfate attack is rarely reported. In fact, when cementitious materials are attacked by sulfate ions, the crack tip will gradually deteriorate, which results in the reduction of the ability to resist hydraulic fracturing. The critical water pressure is a significant parameter, which reflects the driving ability of hydraulic fracturing. Therefore, it is a meaningful work to study the evolution of the critical water pressure of cement mortar under sulfate attack.

The objective of this paper is to fully investigate the influence of sulfate attack on the evolution of hydraulic fracturing in cement mortar via experiment and theory analysis. To accomplish this, a set of cement mortar specimens are immersed in sodium sulfate solution and distilled water (used for reference) firstly. Hydraulic fracturing tests and splitting tensile strength tests are performed at different immersion periods. The variation laws of the critical water pressure and tensile strength of cement mortar immersed in two exposure conditions are also obtained. Secondly, the microstructure development and porosity evolution of the cement mortar subjected to sulfate attack are discussed by using a representative volume element. Based on the three-phase sphere model, the evolution of the tensile strength of cement mortar is also analyzed. Thirdly, based on fracture mechanics, a stress intensity factor at the crack tip of cement mortar under sulfate attack is modified by two proposed damage variables. Meanwhile, the relationship between the critical water pressure and the tensile strength of cement mortar under sulfate attack is established, which is in good agreement with the experimental results. Furthermore, a theoretical model is proposed to describe the evolution of the critical water pressure in cement mortar under sulfate attack. Finally, an empirical formula is suggested to describe the evolution of critical water pressure in cement mortar under sulfate attack.

## 2. Experimental Program

### 2.1. Material and Specimen Preparation

In this paper, all the test specimens are fabricated by using ordinary Portland cement (P.O. 32.5) sourced from Longtan cement plant in Nanjing, China, fine size river sand and local tap water in Nanjing, China. The designed mix proportion of cement, sand, and water is taken as 1:3:0.5 by weight.

**Figure 1:**
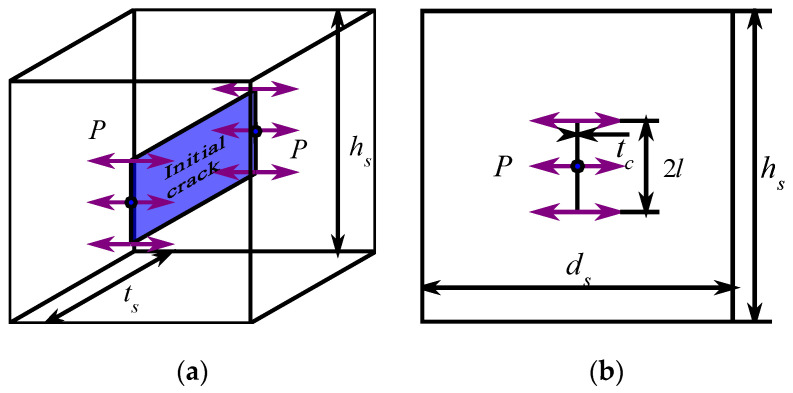
Sketch of cement mortar specimen of hydraulic fracturing test (unit: mm). (**a**) Three-dimensional diagram. (**b**) Front view.

The hydraulic fracturing specimen is designed as a central-precrack cube with a diameter of *h*_s_ × *t*_s_ × *d*_s_ = 150 mm × 150 mm × 150 mm based on [6], which is used for investigating the evolution of the critical water pressure of cement mortar immersed in exposure conditions, as shown in Figure 1. During the central-precrack specimen preparation, a steel plate is buried into the specimen before curing, to precast a crack with the size of 2*l* × *t*_c_ × *t*_s_ = 50 mm × 2 mm × 150 mm in the center position of specimen. In order to easily remove the steel plate from the specimen, demoulding reagent is evenly applied on the steel plate. After the initial setting time of cement mortar, the steel plate is pulled out from the specimen. After 24 h, all specimens are removed from the molds and then cured in water tank for 28 days at a room temperature.

### 2.2. Sulfate Exposure Tests

After 28 days curing, the cement mortar specimens are divided into two groups and denoted by series 1 and series 2 as shown in Table 1. Series 1 is fully immersed in distilled water, which is used for comparison. Series 2 is utilized for analyzing the influence of sulfate attack on the tensile strength and critical water pressure for cement mortar. Actually, the concentration of sulfate ions in aggressive environment is relatively low, and it takes much longer time to obtain corrosion results of cement mortar. In order to shorten the long-term corrosion process of cement mortar, the laboratory-accelerated attack method is utilized in this paper, and the concentration of corrosive ions in solution is increased. According to the studies [26,27], laboratory-accelerated corrosion tests can accelerate the deterioration process of cement mortar and may influence the specific test results, but the deterioration mechanism and variation regularity of the cement mortar under exposure environments are not changed. In view of this, the concentration of ${\mathrm{SO}}_{4}^{2-}$ in sodium sulfate solution is set as 0.1 mol/L in this study. Meanwhile, according to the GB/T 749-2008 [28], the exposure time for specimen can be designed on actual situation. Previous studies [8,29] have shown that the mechanical properties of cement mortar specimens with a water/cement mass of 0.5 experience a decrease stage after about 150 days. Thus, based on previous studies [8,29], the maximum exposure time for the specimen is designed as 210 days in this study, other exposure time is listed in Table 1. Furthermore, the solution is regularly replaced since the over-consumption of corrosive ions in solution may disturb the test results.

### 2.3. Hydraulic Fracturing Tests

**Figure 2:**
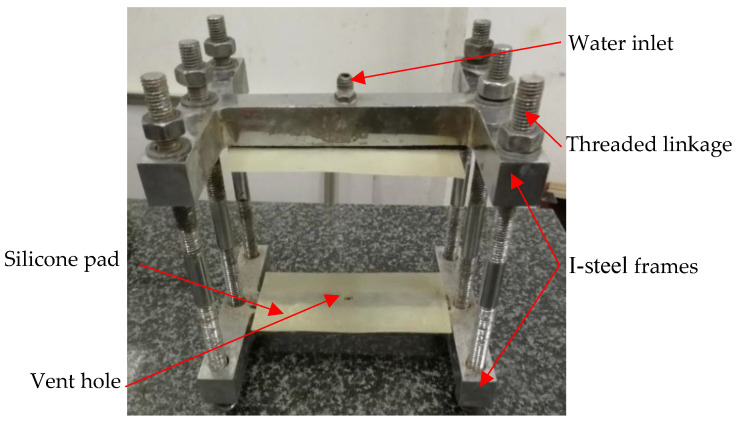
The watertight device.

After being soaked in each exposure conditions, groups of three specimens are removed from solutions with different immersion periods (Table 1). Then, those specimens are applied in hydraulic fracturing tests. Since there are no unified devices or methods for hydraulic fracturing tests of cement mortar, Zhang et al.’s hydraulic fracturing test method and device [6] are chosen in this study. The hydraulic fracturing tests are performed by using a watertight device and hydraulic loading system controlled by a computer. The watertight device, which is shown in Figure 2, mainly includes five parts: a pair of I-steel frames, two silicone pads, six threaded linkages, water inlet and vent hole. In order to make the precast crack propagate through the specimen steadily, and reduce the dispersion of test result, the hydraulic loading speed of the hydraulic loading system is set as 4.7 × 10^−4^ L/s. The procedure of the hydraulic fracturing test mainly includes four steps: (a) place a specimen to the watertight device, and ensure the precrack of the specimen is well connected with the water inlet and vent hole of the watertight device; (b) connect the water inlet of the watertight device together with the hydraulic loading system using a high pressure-resisting pipe; (c) start the hydraulic loading system, exclude the air in the precrack and water pipelines, and then seal the vent hole of the watertight device; (d) load the water pressure until the hydraulic fracturing of the specimen occurs.

### 2.4. Splitting Tensile Strength Tests

According to the method described by the standard DL/T 5150-2017 [30], splitting tensile strength tests are conducted on cement mortar specimens with different immersion time to obtain the evolution of tensile strength. The splitting tensile strength tests are performed on the electro-hydraulic servo universal testing system. In the tests, the applied load is conducted in the stress control mode at a rate of 0.05 MPa/s. The following Equation (1) is used to calculate the tensile strength:

(1) $$f_{t}=\frac{2F}{\pi A}=0.637\frac{F}{A}$$

where $f_{t}$ is the tensile strength (MPa), *F* is the failure load of the cement mortar specimen (N), *A* is the splitting cross-sectional area (m^2^). Three cement mortar specimens from distilled water or sodium sulfate solution are tested at each exposure time (Table 1), respectively, and then the arithmetic average of test results for these three specimens is taken for further analysis.

## 3. Results

### 3.1. Strength Change

**Figure 3:**
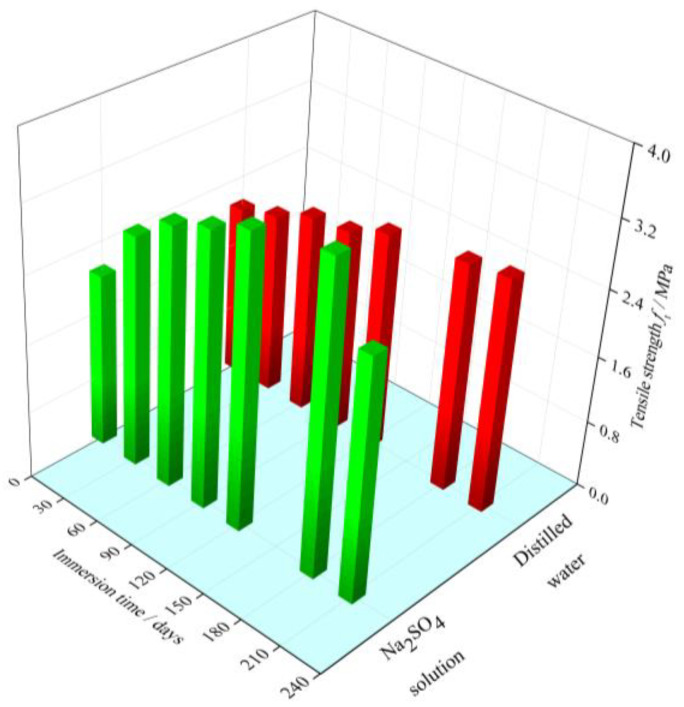
Variation in the tensile strength with immersion time.

The evolution of the tensile strength of cement mortar specimens immersed in the distilled water and sodium sulfate solution for different immersion time is shown in Figure 3. It can be observed that the tensile strength of the specimens exposed to distilled water experiences an obvious increase before early 180 days. After 180 days, there is no significant increase in the tensile strength and its value keeps around 2.75 MPa. This phenomenon may be attributed to the further cement hydration. Previous studies have also indicated that the hydration degree of cement mortar specimens with a water/cement mass ratio of 0.5 in this paper is 70~75% [8,29].

For immersion in the sodium sulfate solution, the cement mortar specimen experiences an apparent increase in tensile strength at the initial stage. However, beyond the immersion time of 180 days, the tensile strength begins to decrease with the increase of immersion time. After 210-day exposure, the tensile strength of the cement mortar specimen decreases by 13.8% in comparison to the peak value of the tensile strength. Moreover, the cement mortar specimens immersed in the sodium sulfate solution have a higher peak value of the tensile strength than that immersed in the distilled water. It is indicated that there are two competitive effects, i.e., enhancement effect and weakening effect, in the evolution of the tensile strength for the cement mortar under sulfate attack.

### 3.2. Evolution of Critical Water Pressure

**Figure 4:**
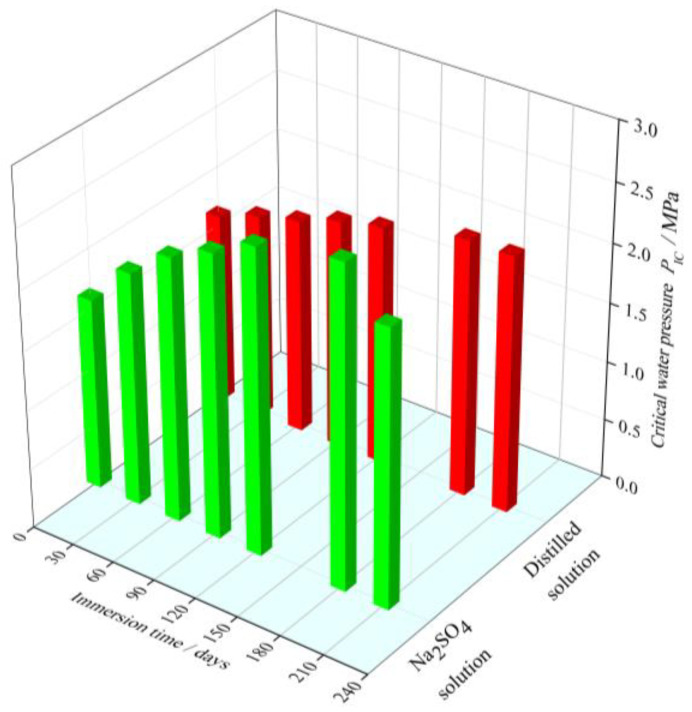
Variation of the critical water pressure with immersion time.

Figure 4 shows the variation of the critical water pressure of the cement mortar specimens exposed to the sodium sulfate solution and distilled water. For the specimens immersed in the distilled water, during the immersion period of 180 days, the critical water pressure of the cement mortar experiences an obvious increase with immersion time, which may be attributed to the further hydration. The unhydrated calcium silicate reacts with the aqueous solution to create the new C-S-H gel, which can fill the pores of cement mortar and improve the density. Therefore, the ability to resist crack propagation in cement mortar under hydraulic pressure is improved. After 180 days, the hydration of the cement mortar is approximately completed and the critical water pressure keeps around 2.17 MPa.

The cement mortar specimens immersed in sodium sulfate solution show a significant difference in the evolution of critical water pressure, compared with that immersed in distilled water. The critical water pressure increases with the immersion time at early stage. After 180 days of the immersion, the cement mortar specimens reach the peak values of the critical water pressure 2.7 MPa. This result may be attributed to the following two reasons. One is that the continuous hydration of cement mortar will improve the density of cement mortar and the ability to resist crack propagation in cement mortar under hydraulic pressure. On the other hand, the sulfate ions invade into the cement mortar, which will react with the pore solution in cement mortar to produce gypsum and ettringite [31,32]. The generated products can assist in filling the original pores of the cement mortar specimens and increase the compactness of structure during the initial immersion period. Therefore, the ability to resist crack propagation in cement mortar under hydraulic pressure increases.

However, beyond the immersion time of 180 days, the critical water pressure of the cement mortar specimens immersed in Na_2_SO_4_ solution begins to decrease. After the 210-day immersion, the critical water pressure of cement mortar is 2.327 MPa, there is a reduction of 13.85% in comparison to the peak value of the critical water pressure. It can be explained by the fact that, with the further development of sulfate attack process, the accumulated sulfate reaction products exceed the accommodating capacity of the pore inside cement mortar, leading to high expansive stress and expansive microcracks. Meanwhile, the formation of a large amount of sulfate reaction products induce the decrease in C-S-H, resulting in the deterioration of the binding performance of cement mortar [33]. Thus, the material structure around the crack-tip will be gradually deteriorated, and the ability to resist crack propagation in cement mortar under hydraulic pressure decreases.

Moreover, it can also be seen that from Figure 3 and Figure 4, under sulfate attack, the critical water pressure displays the similar evolution trend with the tensile strength. The relationship between the critical water pressure and tensile strength under sulfate attack is discussed in following section.

## 4. Theoretical Analysis of Critical Water Pressure under Sulfate Attack

### 4.1. Porosity Development of Cement Mortar under Sulfate Attack

The experimental results indicate that the evolution of the critical water pressure of the cement mortar immersed in sodium sulfate solution is related to the continuous precipitation of hydration and sulfate products. Further, the fundamental reason for the changes of these macroscopic properties lies in the microstructural development inside the cement mortar [33]. Thus, in order to better study the evolution of the critical water pressure under sulfate attack, the porosity development of cement mortar is discussed as follows.

According to Wang and Li’s work [34], a widely used analysis model called representative volume element (RVE) is applied in the present study. Although the RVE is defined as a smallest element, it is the sufficiently large composite element that contains a lot of material information (i.e., pores, microcracks and hydration products) and can represent the effective material properties of the large-scale cementitious material [33,35]. Therefore, we just need to investigate one single RVE for studying the material properties of cement mortar on any scale.

**Figure 5:**
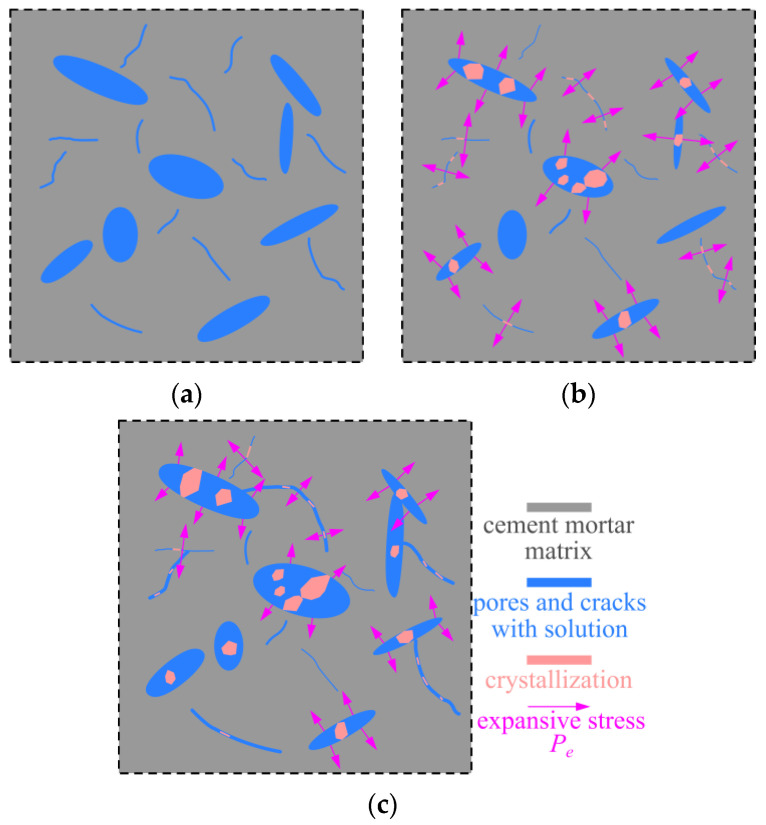
A representative volume element (RVE) for cement mortar in different stages of sulfate attack. (**a**) Cement mortar before sulfate attack. (**b**) The expansive stress appears inside a RVE. (**c**) The expansive stress on some pore surfaces is released.

A RVE of cement mortar before sulfate attack is schematically shown in Figure 5a, which includes pores, microcracks and cement mortar matrix. It should be pointed out that the cement mortar matrix is composed of the cement without complete hydration, hydration products and sand. As discussed previously, the continuously generated hydration products can fill the original pores and refine the microstructure of cement mortar. In this case, the size of pores gradually decreases as well as the porosity *n* in RVE decreases. When the sulfate ions diffuse into cement mortar, which will react with the pore solution in the cement mortar to form sulfate products. These sulfate products and further hydrated products will fill the pores and microcracks of the cement mortar. Thus, the corresponding size of pores and porosity *n* in RVE continually decreases at early stage. Meanwhile, the expansive sulfate products exceed the pore limitation and the expansive stress Pe appears, as shown in Figure 5b. Under this circumstance, the expansive stress *P*_e_ increases with the precipitation of the sulfate products. Once the expansive stress *P*_e_ outstripped the critical value *P*_e_, it will lead to the enlargement of the pore size and the extension of microcracks. This phenomena can result in the increment of the porosity *n* in RVE. Neglecting the interaction effect among pores or microcracks inside the cement mortar, the expansive stress *P*_e_ on the pore surface will be released to zero. At this time, the RVE is depicted in Figure 5c. Moreover, these pores will experience the fill and expansive effect of freshly generated sulfate products again, until they are no longer satisfied the formation condition of expansion stress *P*_e_. Consequently, the evolution of expansive stress *P*_e_ on the surface of pore may be described by a volatility function. The author notices that although the pore-filling effect of expansive sulfate products can occupy the space inside pores, the extension of microcracks is irreversible. The cement hydration will be completed gradually during the later stage of immersion. In addition, the formation of a large amount of sulfate products will induce the decrease in C-S-H, resulting in the exposure of the pores and microcracks that are previously occupied by C-S-H gel. Thus, the porosity *n* in RVE increases during the later stage of immersion.

**Figure 6:**
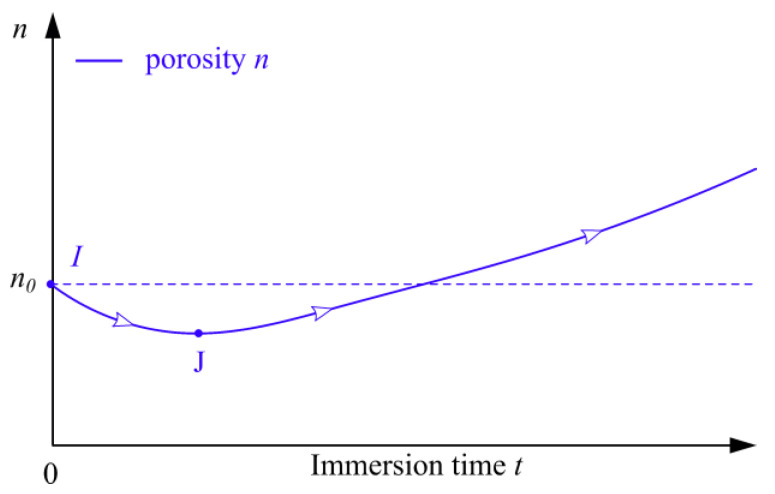
The evolution of the porosity of the RVE subjected to sulfate solution.

In summary, it can be concluded that the porosity *n* of the representative volume element (RVE) under sulfate attack decreases in early immersion period and increases in later period, which is shown in Figure 6. The similar results for the porosity changes of cement mortar subjected to a sulfate environment is also reported in Tekin et al.’s study [36]. Here, the expansive stress of RVE is denoted by symbol “$P_{e}^{*}$”, which is a comprehensive result of expansive stress *P*_e_ on all pores inside RVE. Under sulfate attack, the development of porosity *n* in RVE is closely related to the expansive stress $P_{e}^{*}$. However, we find it difficult to obtain the theoretical value of the expansive stress $P_{e}^{*}$ for RVE at *t* days exposed to a sulfate environment. In view of this, in the following analysis, the effect of the expansive stress $P_{e}^{*}$ on cement mortar is considered to be the development of the porosity *n* of the cement mortar at *t* days in sulfate solution.

### 4.2. Evolution of Tensile Strength under Sulfate Attack

Recently, in order to obtain the relationship between the porosity and the macroscopic effective mechanical properties of concrete, a typical three-phase model was developed [37,38,39]. The three-phase model was used by Duan et al. [40] to investigate the effective shear moduli of the heterogeneous material. Du et al. [35] utilized the three-phase sphere model to analyze the effective mechanical properties of the porous dry concrete. Jin et al. [41] studied the relationship between the porosity and the effective mechanical properties of the saturated concrete by using the three-phase sphere model. Based on the work of previous studies, the three-phase sphere model is adopted to analyze the effective mechanical properties of cement mortar under sulfate attack.

**Figure 7:**
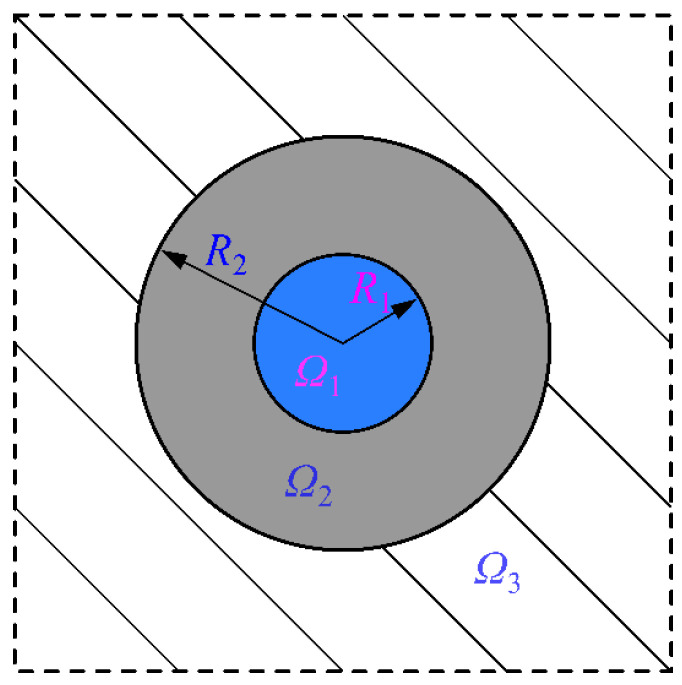
Three-phase sphere model of the cement mortar after the immersion in solution.

This paper considers that the specimens are immersed in solution for a long time before testing, and they are assumed to be saturated. Thus, the three-phase sphere model of cement mortar after immersion in solution is presented in Figure 7. In Figure 7, the region *Ω*_1_ denotes the pore-water phase, the region *Ω*_2_ and *Ω*_3_ refer to the phase of the cement mortar matrix and equivalent homogeneous medium. The radius of the pore-water phase is *R*_1_, and the outer radius of the cement mortar matrix phase is *R*_2_. Then, the volume fraction of the pore-water phase in the cement mortar can be written by $R_{1}^{3}/R_{2}^{3}$, which is equal to the volume fraction of each phase in the cement mortar material [42]. According to the aforementioned porosity development, it can be seen that the radii *R*_1_ and *R*_2_ change with the immersion time during the sulfate attack. It is noticed that the composition and properties of cement mortar matrix will also change with immersion time.

**Figure 8:**
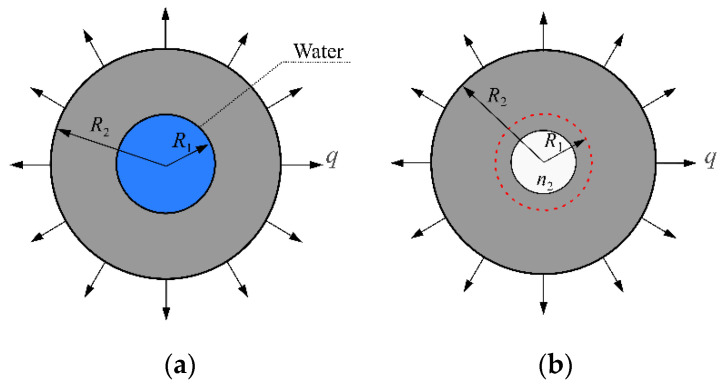
(**a**) A simplified three-sphere model with tensile strength q; (**b**) equivalent hollow sphere model corresponding to (**a**).

When cement mortar materials suffer tensile stress, it is considered that the three-phase sphere model is subjected to a uniform external tensile load *q*. The simplified analysis model under external tensile loading *q* is shown in Figure 8a. The cement mortar matrix and the pore-water are assumed to be isotropic, homogeneous and elastic.

**Figure 9:**
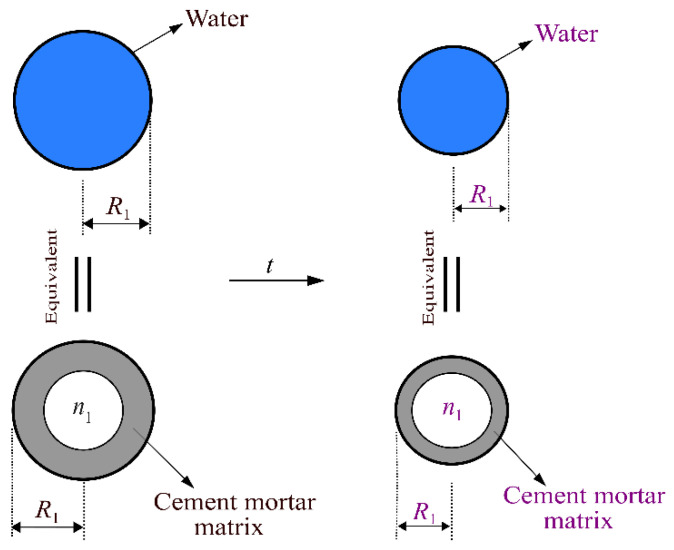
Equivalent process of the spherical pore-water. Different sizes and colors of the radius *R*_1_ represent that the radius *R*_1_ of the spherical pore-water evolves with immersion time.

In order to obtain the stress field of the cement mortar matrix in Figure 8a, the phase of spherical pore-water will be treated as a hollow spherical cement mortar matrix with a porosity *n*_1_ to reflect its mechanical properties [41]. During the equivalent process, the outer radius of the hollow spherical cement mortar matrix and the radius of spherical pore-water are identical and equal to *R*_1_. The equivalent process of spherical pore-water is shown in Figure 9. Then, the equivalent stress analysis model corresponding to Figure 8a is shown in Figure 8b. Thereby, the total porosity *n*_2_ of the hollow cement mortar matrix shown in Figure 8b can be expressed by Equation (2).

(2) $$n_{2}=\frac{R_{1}^{3}n_{1}}{R_{2}^{3}}=nn_{1}$$

**Figure 10:**
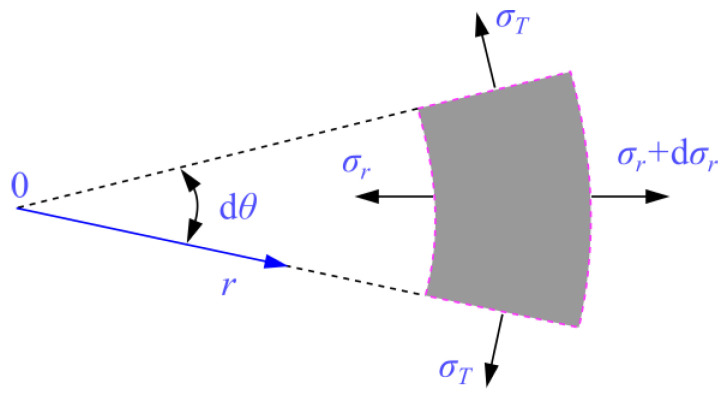
Stress equilibrium analysis of a micro-unit.

The stress equilibrium state of a micro-unit taken out from the hollow sphere model in Figure 8b is shown in Figure 10. In the micro unit, two pairs of circumferential stress acting on radial planes can be represented as $\sigma_{T}$, because of the symmetry. The radial stress acting on the spherical surface and the radial body force within the cement mortar matrix are denoted by $\sigma_{r}$ and $f_{r}$, respectively. Because of the symmetry of the geometric shape and loading condition, there is no shear stress in the micro unit. For convenience, regardless of the radial body force $f_{r}$, the radial stress $\sigma_{r}$ and the circumferential stress $\sigma_{T}$ for cement mortar matrix in Figure 8b can be calculated by Equations (3) and (4), respectively:

(3) $$\sigma_{r}=\frac{q}{1-n_{2}}-\frac{n_{2}R_{2}^{3}q}{1-n_{2}}\frac{1}{r^{3}}$$

(4) $$\sigma_{T}=\frac{q}{1-n_{2}}+\frac{n_{2}R_{2}^{3}q}{2(1-n_{2})}\frac{1}{r^{3}}$$

From Equations (3) and (4), it is easy to see that under the far-field tensile loading *q* (*q* > 0) the circumferential stress $\sigma_{T}$ and the radial stress $\sigma_{r}$ satisfy the relationship in Equation (5).

(5) $$\sigma_{T}>\sigma_{r}$$

Herein, the maximum tensile strength criterion is selected as the failure criterion of the cement mortar matrix under the tensile loading. It is assumed that the cement mortar matrix is always in the elastic state before reaching its strength. Hence, it is not difficult to see that the arrival of the cement mortar matrix strength is determined by the circumferential stress.

In order to analyze the circumferential average stress ${\sigma^{\prime }}_{T}$ of the cement mortar matrix shown in Figure 8a, the analysis model is cut into upper and lower halves. Then, the radial stress transmitted from the external cement mortar to the inner spherical pore-water is given by Equation (6).

(6) $$\sigma_{rw}{|}_{r=R_{1}}=\frac{q}{1-n_{2}}-\frac{qn_{2}R_{2}^{3}}{\left(1-n_{2}\right)}\frac{1}{R_{1}^{3}}=\frac{q}{1-n_{2}}-\frac{qn_{2}}{\left(1-n_{2}\right)}\frac{1}{n}$$

Meanwhile, the upper half of spherical pore-water in Figure 8a is taken out for stress analysis. According to the equilibrium conditions, the circumferential average tensile stress of the spherical pore-water can be expressed by Equation (7).

(7) $${\sigma^{\prime }}_{Tw}=\frac{q}{1-n_{2}}-\frac{qn_{2}}{\left(1-n_{2}\right)}\frac{1}{n}$$

Therefore, the circumferential average stress ${\sigma^{\prime }}_{T}$ within the cement mortar matrix in Figure 8a can be expressed by Equation (8).

(8) $${\sigma^{\prime }}_{T}=\frac{qR_{2}^{2}-{\sigma^{\prime }}_{Tw}R_{1}^{2}}{R_{2}^{2}-R_{1}^{2}}=\frac{qR_{2}^{2}}{R_{2}^{2}-R_{1}^{2}}-\left[\frac{q}{1-n_{2}}-\frac{qn_{1}}{1-n_{2}}\right]\cdot \frac{R_{1}^{2}}{R_{2}^{2}-R_{1}^{2}}$$

When the porosity *n* = 0, the tensile strength of the cement mortar matrix is set as $f_{0}$. It is assumed that the cement mortar matrix works in the linear elastic state before reaching its tensile strength $f_{0}$. Thus, the corresponding peak strain of cement mortar matrix can be given by Equation (9) [35,41].

(9) $$\varepsilon_{0}=\frac{{\sigma^{\prime }}_{T}}{E_{c}}=\frac{q}{E_{c}}=\frac{f_{0}}{E_{c}}$$

where $E_{c}$ is the elastic modulus of the cement mortar matrix.

When the porosity n≠0, the circumferential average strain ${\bar{\varepsilon }}_{c}$ within the cement mortar matrix can be expressed by Equation (10).

(10) $${\bar{\varepsilon }}_{c}=(\frac{R_{2}^{2}}{R_{2}^{2}-R_{1}^{2}}-\frac{1-n_{1}}{1-n_{2}}\cdot \frac{R_{1}^{2}}{R_{2}^{2}-R_{1}^{2}})\cdot \frac{q}{E_{m}}$$

Under the uniform external tensile loading *q*, when the tensile failure of the cement mortar after exposure occurs, the external tensile loading *q* can be regarded as the tensile strength $f_{t}$ of the cement mortar subjected to sulfate attack. Meanwhile, once the cement mortar matrix (n≠0) reaches its tensile strength $f_{0}$, the circumferential average strain ${\bar{\varepsilon }}_{c}$ will be equal to the peak strain $\varepsilon_{0}$ of non-porous cement mortar matrix [35,41]. Therefore, the tensile strength $f_{t}$ of the cement mortar under sulfate attack can be expressed as Equation (11).

(11) $$f_{t}=(1-n^{2/3})\cdot \frac{1-n_{2}}{n_{1}-n_{2}}\cdot f_{0}$$

It can be seen from Equation (11) that $f_{t}$ is related to the porosity and the *f*_0_. According to the above analysis, the porosity *n* of cement mortar specimens immersed in sulfate solution shows a decrease during the early immersion period, while *f*_0_ remains invariant. Besides, in the later stage, as the immersion time increases, the porosity *n* of cement mortar increases, and *f*_0_ decreases. Therefore, it can be seen from Equation (11) that the tensile strength of the cement mortar under sulfate attack increases in the early immersion period and decreases in the later immersion period, which is consistent with the test results of the tensile strength.

### 4.3. Evolution Model of Critical Water Pressure under Sulfate Attack

According to the above analysis, the sulfate attack can lead to the extension of microcracks and induce the deterioration of the binding performance of cement mortar. Therefore, for the cement mortar specimen with a crack, the crack length and fracture stress can be affected by sulfate attack. Based on these two changeable characteristics, the evolution model of critical water pressure can be obtained in this paper.

For convenience, the hydraulic fracturing specimen is simplified as a rectangle plate with a central crack in this paper. According to the theory of fracture mechanism, when the hydraulic pressure acts on the precrack surface in the specimen, the stress intensity factor at the crack tip of the uncorroded hydraulic fracturing specimen can be written as Equation (12) [43,44].

(12) $$K=\beta P\sqrt{\pi d}$$

where K is the stress intensity factor at the crack tip, *P* is the hydraulic pressure on the crack surface of the specimen, β is the parameter, which is related to the specimen geometry shape and the crack shape, and d is the effective crack length.

For the uncorroded specimen, the criterion of the crack propagation failure can be expressed as Equations (13) and (14) [44].

(13) $$K=K_{IC}$$

(14) $$K_{IC}=\sigma_{f}\sqrt{\pi d}\varphi$$

where $K_{IC}$ is the fracture toughness of the cement mortar specimen, $\sigma_{f}$ is the fracture stress of the cement mortar specimen, and φ is a parameter, which is related to the crack shape and loading mode.

In this paper, two influential factors of sulfate attack on critical water pressure, i.e., the variation of the crack length and fracture stress are considered. Then, the stress intensity factor at the crack tip and fracture toughness under sulfate attack can be written as Equations (15) and (16), respectively.

(15) $$K=\beta P\sqrt{\pi (1+\omega )d}$$

(16) $$K_{IC}=\sigma_{f}(1-\lambda )\sqrt{\pi d}\varphi$$

where ω and λ are the damage variables of the crack length and fracture stress under sulfate attack, respectively, expressed as Equations (17) and (18).

(17) $$\omega =\frac{d_{t}-d}{d}$$

(18) $$\lambda =\frac{\sigma_{f}-\sigma_{f,t}}{\sigma_{f}}$$

where $d_{t}$ and $\sigma_{f,t}$ represent the effective crack length and fracture stress of the specimen under sulfate attack, respectively.

When the hydraulic fracturing occurs in the cement mortar specimen immersed in sulfate solution, the stress intensity factor at the crack tip K in Equation (15) will be equal to the fracture toughness of the cement mortar specimen $K_{IC}$ in Equation (16). Thus, Equation (19) is obtained.

(19) $$\beta P\sqrt{\pi d}\sqrt{1+\omega }/(1-\lambda )=\sigma_{f}\sqrt{\pi d}\varphi$$

According to the definition of the stress intensity factor at the crack tip, from the left hand of Equation (19), it is easy to see that the stress intensity factor $K^{\prime }$ of cement mortar specimen under sulfate attack can be described by Equation (20).

(20) $$K^{\prime }=\sqrt{1+\omega }/(1-\lambda )\beta P\sqrt{\pi d}=\psi \beta P\sqrt{\pi d}$$

where ψ is the damage coefficient of the cement mortar specimen under sulfate attack, which is expressed as Equation (21).

(21) $$\psi =\frac{\sqrt{1+\omega }}{1-\lambda }$$

It can be seen from the above derivation process that the stress intensity factor in Equations (19) and (20) take into account the increase of crack length and the deterioration of material property under sulfate attack. These two factors are equivalent to the damage coefficient of the cement mortar specimen with a crack, so the length crack of the original crack and the fracture toughness are still available. In view of this, Equation (20) can be regarded as the modified stress intensity factor at the crack tip of the cement mortar specimen under sulfate attack.

Many researches [45,46] show that one of the fundamental reasons for unstable failure of cement mortar materials is that its stress concentration of crack tip arrives tensile strength, which leads to the crack propagation in cement mortar materials. Thus, Equation (22) is obtanined.

(22) $$\sigma_{f}=f_{t}$$

Then, combining Equations (19), (21) and (22), gives the relationship between the critical water pressure and tensile strength as Equation (23).

(23) $$\left\{ \begin{array}{l} P_{IC}=\eta f_{t}\\ \eta =\frac{\varphi }{\beta \psi } \end{array}\right.$$

where the coefficient η reflects the relation between the critical water pressure and the tensile strength under sulfate attack. It should be noted that the parameters β and φ can be determined according to the handbook of stress intensity factors. The damage variables of the crack length and fracture stress evolve with the immersion time.

**Figure 11:**
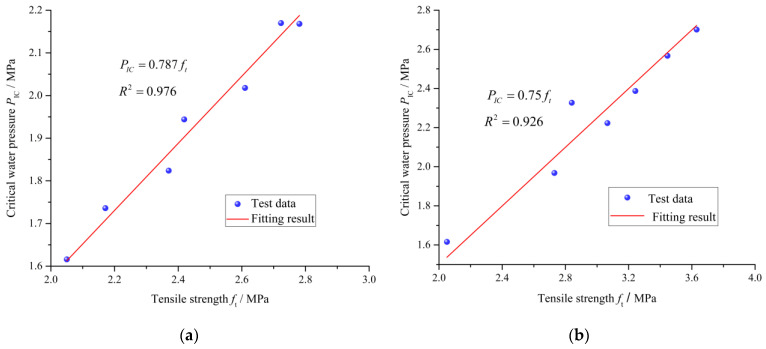
Relationship between the critical water pressure and the tensile strength of the cement mortar immersed in different exposure conditions. (**a**) Distilled water. (**b**) Sodium sulfate solution.

As shown in Figure 11, the experimental relationship between the critical water pressure and the tensile strength of the cement mortar in exposure conditions is further analyzed. It can be seen that the square of correlation coefficients *R*^2^ are more than 0.92.

This indicates that the immersion time has no significant impact on the coefficient η in Equation (23). It is suggested that there is a good linear relationship between the critical water pressure and the tensile strength of the cement mortar under exposure conditions.

Substituting Equation (23) into Equation (11) gives the evolution model of the critical water pressure in the cement mortar under sulfate attack as Equation (24).

(24) $$P_{IC}=\eta (1-n^{2/3})\cdot \frac{1-n_{2}}{n_{1}-n_{2}}f_{0}$$

The Equation (24) shows that the critical water pressure of the cement mortar under sulfate attack is related to the porosity *n* and the cement mortar matrix tensile strength *f*_0_. Similar to the analysis for tensile strength in Section 4.2, during the early stage of sulfate attack, the porosity *n* of cement mortar decreases, and the matrix tensile strength *f*_0_ remains invariant. For the sulfate attack progress, at the later stage, the porosity *n* increases, and the cement mortar matrix tensile strength *f*_0_ decreases. Therefore, it can be seen from Equation (24) that the critical water pressure of the cement mortar under sulfate attack increases in the early immersion period and decreases in the later immersion period, which is consistent with the test results of the critical water pressure.

Although the evolution of the critical water pressure for the cement mortar under sulfate attack can be determined by the Equation (24), it is regrettable that the porosity and matrix tensile strength of cement mortar under sulfate attack are not measured in this study. Therefore, the evolution of the critical water pressure for the cement mortar with immersion time cannot be directly determined in this study.

Hence, an indirect method is proposed to describe the evolution of critical water pressure of cement mortar under sulfate attack. In this method, a non-dimensional critical water pressure $P_{IC}^{*}$, is defined, which is given by Equation (25).

(25) $$P_{IC}^{*}=\frac{P_{IC}}{P_{IC0}}$$

where $P_{IC}^{*}$ is the relative critical water pressure of the cement mortar specimen, $P_{IC}$ and $P_{IC0}$ are the critical water pressure of the cement mortar immersed in exposure environment for *t* days and 0 day, respectively.

**Figure 12:**
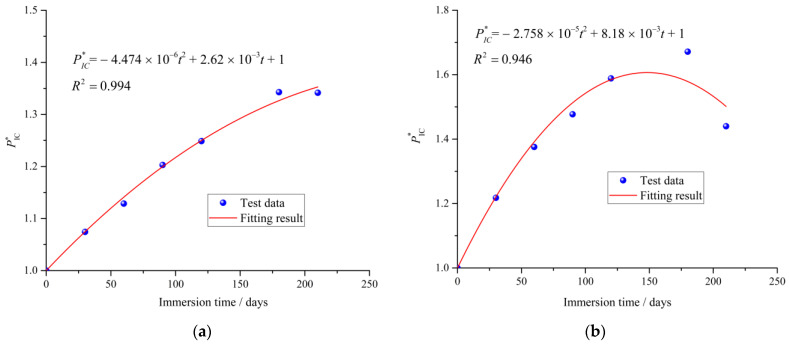
Fitting results of the critical water pressure for different exposure conditions. (**a**) Distilled water. (**b**) Sodium sulfate solution.

According to the experimental results shown in Figure 4, the evolution of the critical water pressure of the cement mortar exposed to sulfate solution exhibits two obvious stages, i.e., an initial increase stage and a subsequent decrease stage. Therefore, the most likely simple form of $P_{IC}^{*}$ versus immersion time may be a binomial function, which is presented as Equation (26).

(26) $$P_{IC}^{*}=at^{2}+bt+1$$

where the parameters *a* and *b* are determined by using a curve-fitting method with experimental results. As shown in Figure 12, it can be seen that the proposed empirical formulas in Equation (26) are acceptable. Due to the immersion time series is not long enough in this study, the Equation (26) is used only for effectively describing the increase stage of critical water pressure of cement mortar exposed to distilled water.

## 5. Conclusions

In this paper, the hydraulic fracturing of the cement mortar under sulfate attack is investigated by experiment and theory analysis. Under sulfate attack, the critical water pressure of cement mortar increases during the initial stage of the immersion, after that, a decrease stage is found. In addition, the critical water pressure and the tensile strength have a similar variation law. According to the fracture mechanics, a stress intensity factor is modified by two proposed damage variables which are crack length and fracture stress. Based on the proposed stress intensity factor, the linear relationship between the tensile strength and the critical water pressure of the cement mortar under sulfate attack is established. An evolution model of the critical water pressure in the cement mortar under sulfate attack is also proposed on the basis of the proposed model of tensile strength. It is found that the matrix tensile strength and porosity of cement mortar strongly affect the evolution of the critical water pressure under sulfate attack. Moreover, an empirical formula is suggested to describe the variation law of critical water pressure in the cement mortar under sulfate attack, and its validity is verified by experimental results.

## Figures and Tables

**Table 1:** Two different exposure environments (mol/L).

Project	Exposure Condition	Specimen Grouping	Exposure Time (Days)
Series 1	Distilled water	Three specimens in a group, 7 groups	0, 30, 60, 90, 120, 180, 210
Series 2	Na_2_SO_4_ solution	Three specimens in a group, 7 groups	0, 30, 60, 90, 120, 180, 210

## Data Availability

The data presented in this study are available upon request from the corresponding author.
